# MicroRNA-210 Regulates Mitochondrial Free Radical Response to Hypoxia and Krebs Cycle in Cancer Cells by Targeting Iron Sulfur Cluster Protein ISCU

**DOI:** 10.1371/journal.pone.0010345

**Published:** 2010-04-26

**Authors:** Elena Favaro, Anassuya Ramachandran, Robert McCormick, Harriet Gee, Christine Blancher, Meredith Crosby, Cecilia Devlin, Christopher Blick, Francesca Buffa, Ji-Liang Li, Borivoj Vojnovic, Ricardo Pires das Neves, Peter Glazer, Francisco Iborra, Mircea Ivan, Jiannis Ragoussis, Adrian L. Harris

**Affiliations:** 1 Genomics Group, Wellcome Trust Centre for Human Genetics, University of Oxford, Oxford, United Kingdom; 2 Molecular Oncology Laboratories, Weatherall Institute of Molecular Medicine, University of Oxford, John Radcliffe Hospital, Oxford, United Kingdom; 3 Department of Therapeutic Radiology, Yale University School of Medicine, New Haven, Connecticut, United States of America; 4 Indiana University, Indianapolis, Indiana, United States of America; 5 Gray Institute for Radiation Oncology & Biology, University of Oxford, Oxford, United Kingdom; 6 Molecular Haematology Laboratories, Weatherall Institute of Molecular Medicine, University of Oxford, John Radcliffe Hospital, Oxford, United Kingdom; University of Illinois at Chicago (UIC), United States of America

## Abstract

**Background:**

Hypoxia in cancers results in the upregulation of hypoxia inducible factor 1 (HIF-1) and a microRNA, hsa-miR-210 (miR-210) which is associated with a poor prognosis.

**Methods and Findings:**

In human cancer cell lines and tumours, we found that miR-210 targets the mitochondrial iron sulfur scaffold protein ISCU, required for assembly of iron-sulfur clusters, cofactors for key enzymes involved in the Krebs cycle, electron transport, and iron metabolism. Down regulation of ISCU was the major cause of induction of reactive oxygen species (ROS) in hypoxia. ISCU suppression reduced mitochondrial complex 1 activity and aconitase activity, caused a shift to glycolysis in normoxia and enhanced cell survival. Cancers with low ISCU had a worse prognosis.

**Conclusions:**

Induction of these major hallmarks of cancer show that a single microRNA, miR-210, mediates a new mechanism of adaptation to hypoxia, by regulating mitochondrial function via iron-sulfur cluster metabolism and free radical generation.

## Introduction

Hypoxia is a major physiological difference between tumours and normal tissue, mainly generated by tumour growth with inadequate blood supply and consumption of oxygen by tumour cells [reviewed in [1]]. Hypoxia induces a complex transcriptional response mainly via induction of hypoxia inducible factor 1α (HIF1α), affecting many biological processes such as the glycolytic pathway, angiogenesis, pH regulation, invasion and immortalisation [2].

An emerging paradigm in hypoxia is that mitochondria produce reactive oxygen species, mediated by electron transport continuing in hypoxia [3]. This free radical pathway contributes to upregulation of HIF [4] and enhanced growth in vivo [5], yet may also be toxic. A variety of pathways induced by HIF have already been reported to protect from the latter effect, for example induction of pyruvate dehydrogenase kinase inhibits the enzyme complex of pyruvate dehydrogenase and blocking the conversion of pyruvate to acetyl coenzyme A, the first step in the Krebs cycle [6] and enhances lactate production [7]. Mitophagy can be induced by the BH3 domain protein BNIP3, [8], and cytochrome C oxidase subunits may switch to more efficient ones [9].

Recently microRNAs (miRs), which are small (∼22 nt) non-coding RNAs that regulate post-transcriptional gene expression by blocking translation of target mRNAs or by accelerating their degradation [10], [11], have been reported to be induced by hypoxia. However, few of their targets or mechanisms of action are known [reviewed in [12]]. We and others [13] have shown miR-210 is robustly induced by hypoxia in many cell lines, via HIF1α [14]. We recently analysed its expression in a series of 216 breast cancer patients and showed miR-210 expression was correlated with many HIF1α targets at mRNA level (as measured by a hypoxia metagene) and was strongly associated with poor patient survival. Derived only from sequence-based algorithms, some of the previously validated targets of miR-210 include Ephrin A3 [15], E2F3 [16], RAD52 [17], CASP8AP2 [18], and MNT [19]. We combined publically available algorithms, with our gene array datasets, to predict potential miR targets of importance in cancer cells. We found that the mitochondrial iron sulfur cluster homologue ISCU was the highest predicted target for miR-210. Recently, ISCU has been identified as a miR-210 target also in normal pulmonary endothelial cells [20], where it contributes to the Pasteur effect and controls the level of ROS production in hypoxia, suggesting its potential adaptive role to hypoxia in the context of pulmonary endothelium.

Iron sulfur clusters [Fe-S] are present in the active sites of many enzymes and proteins, critical for their activity and capable of conferring regulation by redox status [21]. These clusters are assembled in mitochondria [22] by a complex series of chaperones and enzymes including ISCU, then exported to the cytoplasm, where they are assembled into the relevant protein [23]. Amongst the Fe-S cluster proteins involved are several that comprise key components of complex I, II and III in the mitochondria, and components of the Krebs cycle such as succinate dehydrogenase and aconitase. The cytoplasmic form of the latter regulates iron metabolism via its function as a translational regulator-IRP1 [24].

In this report we show the major biological effects of miR-210 targeting ISCU, all of which are likely to contribute to important phenotypes in cancer. By downregulating ISCU, miR-210 decreases Krebs cycle enzyme activity and mitochondrial function, provides a major mechanism for the increased free radical generation in hypoxia, increases cell survival under hypoxia, induces a switch to glycolysis in normoxia and hypoxia (Warburg and Pasteur effects) and upregulation of the iron uptake required for cell growth. Importantly, analysis of over 900 patients with different tumour types showed that suppression of ISCU is strongly correlated with a worse prognosis. This study thus reveals a new pathway activated in hypoxic tumours, mediated by miR-210 affecting mitochondrial enzyme activity and free radical generation and highlights the importance of mitochondrial metabolism in hypoxia biology [3].

## Results

### Selection of ISCU as a potential target

We compared miR-210 expression in our published series of breast cancer [14] with our hypoxia metagene of clustered mRNAs [25] and combined assessment with target prediction algorithms showed ISCU was the highest predicted target, and three known target genes also sit in highly-ranked positions were selected by this approach (Supporting Material and Methods S1, Supporting Table S1).

### MiR-210 and ISCU mRNA undergo reciprocal regulation

**Figure 1:**
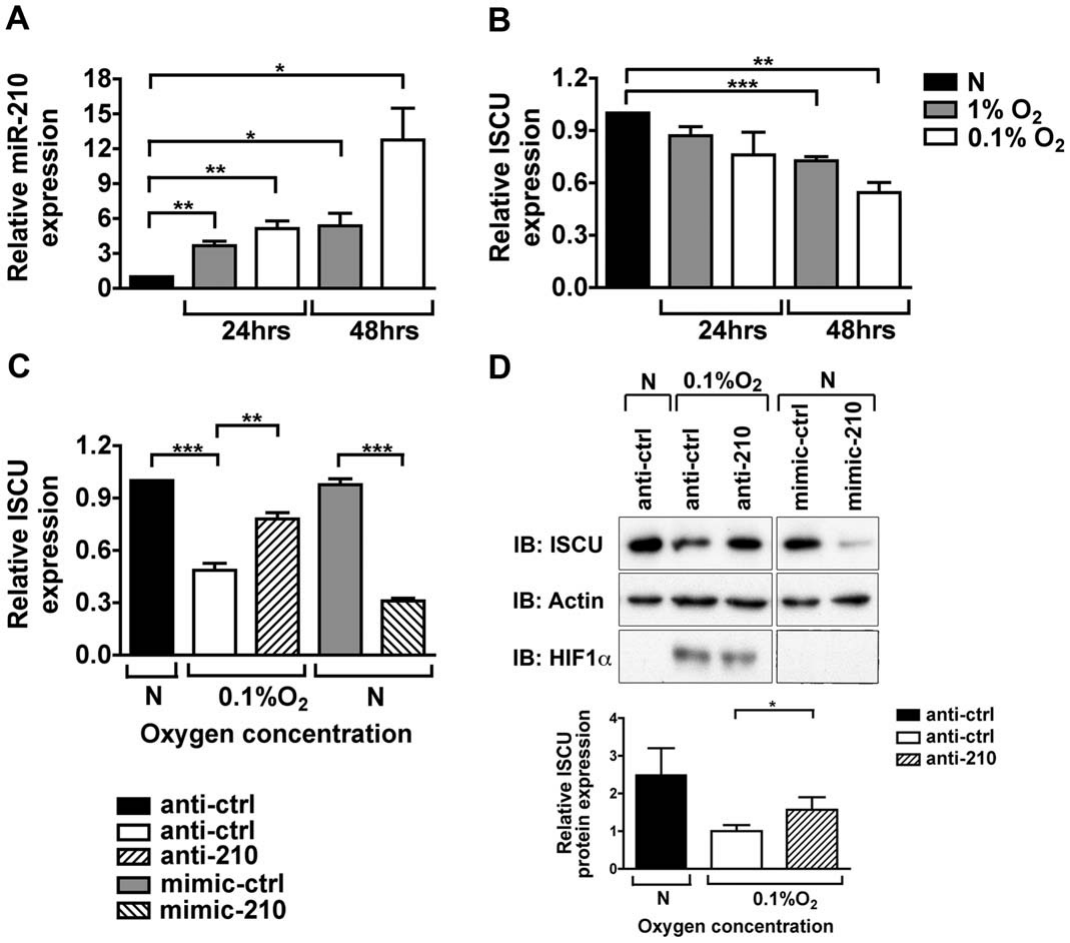
MiR-210 regulates ISCU expression in MCF7 cells. *A*, miR-210 increases under hypoxia, with the most robust induction seen at 48 hrs with 0.1% oxygen. *B*, under the same conditions, the strongest downregulation of ISCU mRNA is observed at 48 hrs with 0.1% oxygen. The expression levels of miR-210 and ISCU mRNA under hypoxia are relative to their normoxic expression levels at 24 hrs (N). Mean ± s.e.m. of three independent experiments is shown (* p<0.05, ** p<0.01, *** p<0.001). *C*, transfection of anti-210 partially rescues the hypoxic suppression of ISCU mRNA and mimic-210 overexpression decreases ISCU mRNA in normoxia at 48 hrs. Expression of ISCU mRNA is relative to the anti-ctrl in normoxia (N). Mean ± s.e.m. of three independent experiments is shown (** p<0.01, *** p<0.001). *D*, (*top panel*) ISCU protein is downregulated in MCF7 lysates at 0.1% oxygen compared to normoxic lysates (N) when the anti-ctrl is transfected for 48 hrs. This decrease in protein level under hypoxic conditions is partially rescued when the anti-210 is transfected. Under normoxic conditions, mimic-210 suppresses ISCU protein levels compared to mimic-ctrl transfected cells. *D*, (*bottom panel*), quantification of the rescue of ISCU protein level upon transfection of anti-210. Transfection of anti-210 rescues the ISCU protein levels in MCF7 by approximately 40%. Mean ± S.E.M of two independent experiments is shown. (* p<0.05).

In agreement with previous publications [13], [14], miR-210 was robustly induced in MCF7 and HCT116 by hypoxia (1% or 0.1% oxygen) at 24 and 48 hrs, with maximal induction observed at 48 hours in 0.1% oxygen (Figure 1A and Supporting Figure S1A). ISCU mRNA quantified by RTPCR was inversely correlated to miR-210 and decreased most significantly when cells were exposed to more severe hypoxia for longer periods (Figure 1B and Supporting Figure S1B). Similar to the regulation of miR-210 under hypoxia, ISCU suppression at the transcript level was dependent on HIF1α and not HIF2α (Supporting Figures S1C and S1D). Furthermore, the phenomenon of reciprocal regulation of miR-210 and ISCU by hypoxia was also found in many other cell lines (Supporting Figures S1E and S1F).

### Mimic-210 suppressed ISCU in normoxia and anti-210 reversed the suppression in hypoxia

We recapitulated the observed hypoxic induction of miR-210 by transfecting MCF7 and HCT116 with mimic-210 in normoxia. Mimic-210 suppressed ISCU mRNA levels by approximately 60% (Figure 1C and Supporting Figure S2A). We then antagonised miR-210 induced under hypoxic conditions with anti-210. Inhibition of endogenous miR-210 significantly rescued the suppression of ISCU mRNA, although this was not complete (Figure 1C and Supporting Figure S2A).

ISCU has two main alternatively spliced isoforms. ISCU1 has an alternative N-terminal sequence and is localised to the cytosol, while ISCU2 is associated with the mitochondria. To confirm the specificity of the antibody used in these studies, MCF7 cells were treated with siRNAs against ISCU. The band detected in the control cells was completely suppressed upon transfection with siISCU1 and siISCU3 (Supporting Figure S2B). The two isoforms of ISCU could not be distinguished by Western blotting of whole cell lysates because of the minor difference in molecular weight. However, the immunoreactive band was detected in both the cytosolic and mitochondrial fractions (Supporting Figure S2C). This band is referred to as ISCU protein and its molecular weight is compatible with the results of Tong et. al. [23].

Under hypoxia, MCF7 and HCT116 cells showed a reduction in ISCU protein, and this was replicated with mimic-210 in normoxia (Figure 1D and Supporting Figure S2D). Moreover, anti-210 partially reversed the hypoxic suppression of ISCU protein (Figure 1D and Supporting Figure S2D). Hypoxia strongly suppressed a luciferase reporter containing the 3′UTR of ISCU that included the putative miR-210 target site and this suppression could be partially rescued by transient transfection of anti-210 (Supporting Figure S2E). In addition, mimic-210 substantially suppressed the luciferase activity compared to the control in normoxic MCF7 cells (Supporting Figure S2E). Finally, while siRNA against ISCU led to a downregulation of both endogenous ISCU and an exogenous tagged ISCU lacking the 3′UTR, mimic-210 mediated downregulation was only observed on the endogenous ISCU protein (Supporting Figure S2F). Taken together, these observations demonstrate that miR-210 downregulates ISCU mRNA and protein levels by targeting its 3′UTR.

### Effects of ISCU downregulation on Fe-S proteins

**Figure 2:**
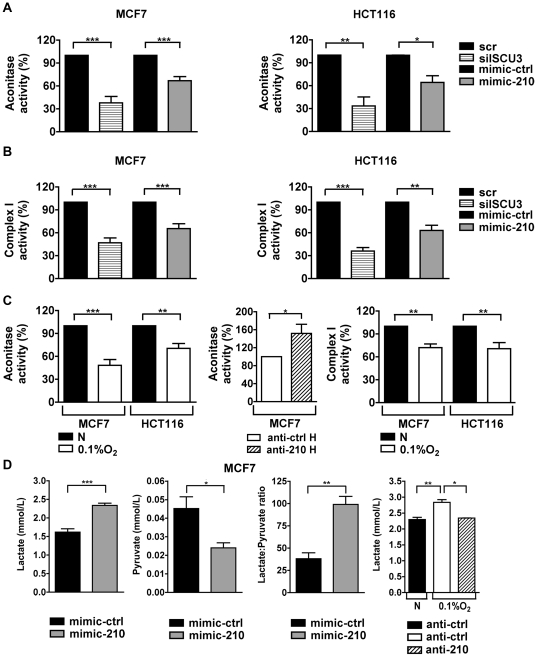
MiR-210 negatively regulates aconitase and complex I activity in MCF7 and HCT116, and upregulates glycolysis. Cells transfected with siRNA against ISCU or with mimic-210 show a significant decrease in the activity of the Fe-S protein aconitase (*A*) and complex I (*B*) at 48 hrs. Activity is expressed relative to scr for siISCU3 or to mimic-ctrl for mimic-210. Mean ± s.e.m. of three independent experiments is shown (* p<0.05, ** p<0.01, *** p<0.001). *C*, hypoxia decreases aconitase and complex I activity in MCF7 and HCT116. Activity is expressed relative to normoxia (N). Transfection of MCF7 with anti-210 increases the level of aconitase activity, compared to anti-ctrl. Mean ± s.e.m. of three independent experiments is shown (* p<0.05, ** p<0.01, *** p<0.001). *D*, in normoxia, MCF7 transfected with mimic-210 have an increase in the secreted lactate concentration and a reduction in the extracellular pyruvate after 48 hours. Lactate: Pyruvate ratio shows a significant increase under the same conditions. Transfection with anti-210 in hypoxia reduced lactate production compared to anti-ctrl transfected cells. Mean ± s.e.m. of three biological replicates is shown (* p<0.05, ** p<0.01, *** p<0.001).

An expected effect of downregulation of ISCU would be a reduction of Fe-S delivery to target proteins. Therefore we analysed the impact of ISCU repression on two enzymes that require Fe-S for their activities, aconitase and mitochondrial complex I. siRNA against ISCU significantly reduced aconitase activity in MCF7 and HCT116 cells compared to control cells in normoxia (Figure 2A). A similar decrease in aconitase activity was evident in both cell lines upon transfection of mimic-210 (Figure 2A). However there was no change in aconitase protein levels as assessed by Western blot (Supporting Figure S3A). Aconitase depleted of Fe-S acts as the translational regulator-IRP1, to increase uptake of iron. We found the mimic-210 increased iron uptake in HCT116 cells (Supporting Figure S3B). There was also clear inhibition of mitochondrial complex I activity induced by mimic-210, in the two cell lines (Figure 2B). In both cell lines there was down regulation of activity by hypoxia, to a similar extent to that induced by mimic-210 or by ISCU depletion. Moreover, transfection of anti-210 in MCF7 cells was able to significantly increase aconitase activity in hypoxia (Figure 2C).

As the decrease in aconitase and complex I activity reduces the Krebs cycle and mitochondrial function we investigated whether there was a shift to glycolysis and lactate production. Our results showed a highly significant reduction of pyruvate and increase in lactate in normoxia with the mimic-210, with an increase in lactate pyruvate ratio. There was also a decrease in lactate production with the anti-210 in hypoxia (Figure 2D).

### Effects of ISCU downregulation on free radical production

**Figure 3:**
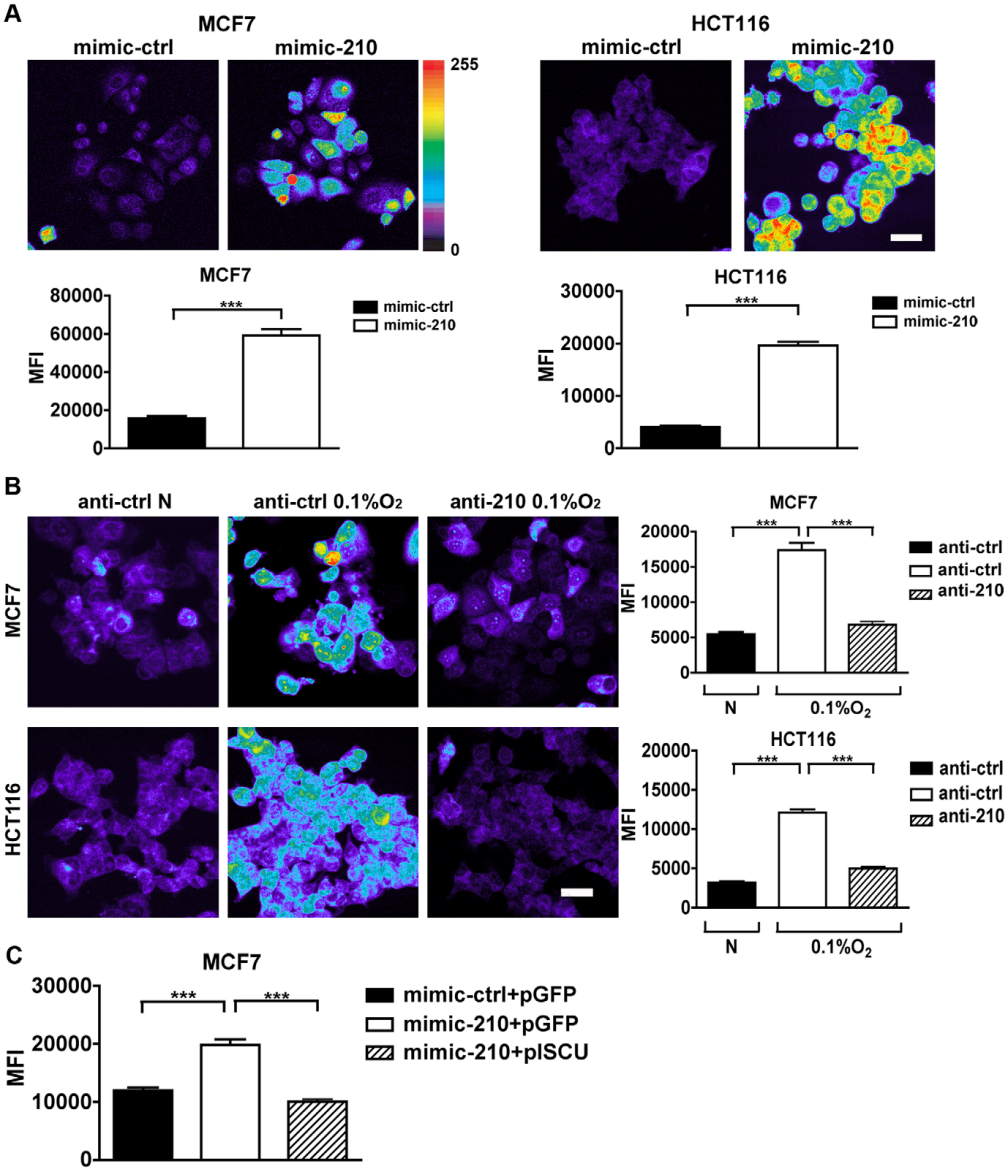
MiR-210 increases the formation of ROS in MCF7 and HCT116 cell lines. *A*, in normoxia, transfection of mimic-210 significantly increases superoxide production at 48 hrs as measured by MitoSox staining. Representative images from mimic-210 transfected cells are shown on the top panels and the mean ± s.e.m. of approximately 300 cells are shown on the bottom panels (***, p<0.001). *B*, exposure of cells to 0.1% oxygen for 48 hrs enhances the production of superoxide; this effect is almost completely reversed upon transfection of anti-210. Representative images from miR-210 transfected are shown on the left panels and the mean ± s.e.m. of approximately 300 cells are shown on the right panels (***, p<0.001). *C*, ROS production induced by mimic-210 is inhibited in MCF7 cells by cotrasfection with the ISCU2 expressing plasmid. Mean ± s.e.m. of approximately 300 cells is shown (*** p<0.001). Bars  = 10 µm.

The loss of Fe-S from complex I is likely to affect the transport of electrons in the electron transport chain and impact on free radical production. We therefore measured the production of superoxide in MCF7 and HCT116 cells. In normoxia, both cell lines showed a marked increase in free radical production upon transfection of mimic-210 compared to mimic-control (Figure 3A).

In hypoxia we noted a highly significant increase in superoxide production, which was nearly completely reversed by anti-210 (Figure 3B). Additionally, transfection of the ISCU2 construct nearly completely reversed the free radical induced by miR-210 (Figure 3C). This demonstrates a major new mechanism for regulation of ROS in hypoxia, and that it is not a passive effect of reduced oxygen availability.

### Effects of miR-210 on apoptosis and survival in normoxia and hypoxia

**Figure 4:**
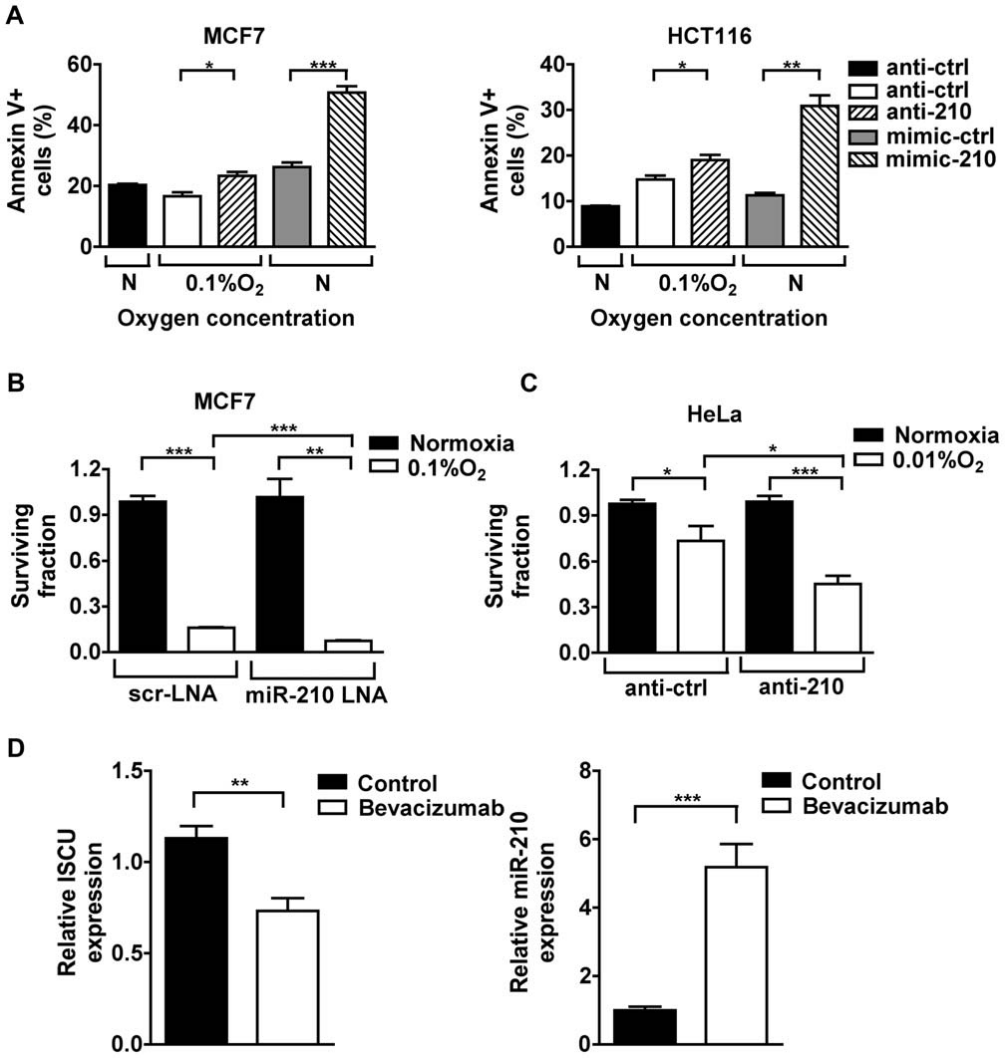
MiR-210 affects cell survival and is inversely correlated to ISCU in hypoxic tumor xenografts. *A*, apoptosis (Annexin V+ cells) was measured in MCF7 (*left*) and HCT116 (*right*) cells treated with miR-210 inhibitor or mimic, after 48 hours exposure to 0.1% oxygen or normoxia. Mean ± s.e.m. is representative of 3 independent experiments (* p<0.05, ** p<0.01, *** p<0.001). *B*, after transfecting MCF7 cells with scrambled LNA or mir-210 LNA and exposed to 0.1% oxygen or normoxia (48 hrs), the cells were re-plated (100 and 500 cells/well). After a 12-day incubation, colonies were fixed, stained, and the surviving fractions were calculated based upon the plating efficiency. *C*, surviving fractions were calculated as in (*B*) in HeLa cells, which were transfected with anti-ctrl or anti-210 (Applied Biosystems/Ambion, Austin, TX, USA) and exposed to 0.01% oxygen or normoxia. In all colony assays, mean ± s.e.m. is representative of 2 independent experiments carried out in triplicate employing 2 different plating densities. (** p<0.01, *** p<0.001). *D*, RT-PCR for ISCU and miR-210 in U87 xenografts treated with anti-angiogenic therapy (bevacizumab). Relative expression of ISCU normalised to β-actin, miR-210 normalised to three small nucleolar controls, RNU43, RNU44 and RNU48. Mean expression ± s.e.m. in 4 animals/group is shown (** p<0.01, ***p<0.001).

Previous studies in yeast have shown the lethal consequences of the ISCU homologs deletion [23]. This notion led us to investigate the effects of miR-210 on apoptosis in normoxia and hypoxia. There was a striking difference in the effects of mir-210 in these opposing conditions. In normoxia, mimic-210 substantially increased apoptosis as measured by annexin V staining, but in hypoxia, antagonism of miR-210 increased apoptosis (Figure 4A).

To evaluate effects of miR-210 on cell proliferation under hypoxic conditions, a clonogenic assay was used with a locked nucleic acid antagonist (LNA) miR-210 molecule (Figure 4B). Although most of the reduction in clonogenicity in hypoxia is clearly via other mechanisms, the effects of the anti-210 resulted in decreased clonogenic survival in hypoxia, which we also found in a more hypoxia-resistant cell line, HeLa cells (Figure 4C).

### In vivo suppression of ISCU by hypoxia and clinical significance of ISCU expression

We investigated whether expression of ISCU gene expression was regulated in vivo, by studying human tumour xenografts. Xenografts of the glioblastoma cell line U87 were treated with the VEGF inhibitor Avastin (Bevacizumab), or with vehicle control. Immunohistochemistry of these tumours demonstrated Avastin-induced necrosis, expression of HIF1α and the HIF target genes CA9 and VEGF (data not shown). We analysed the mRNA from the tumours and found marked upregulation of miR-210 and reciprocal downregulation of ISCU mRNA (Figure 4D).

**Figure 5:**
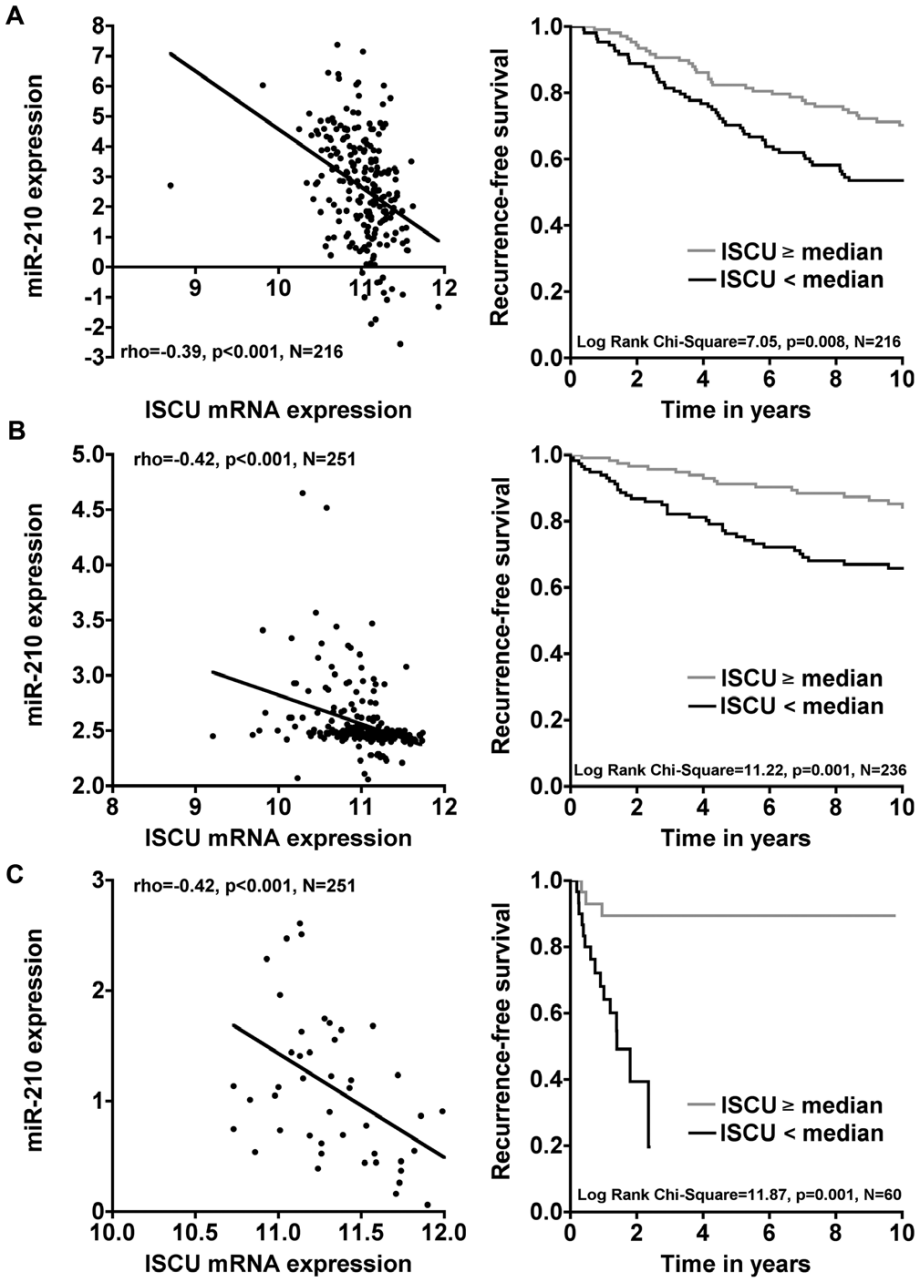
ISCU suppression is associated to miR-210 over-expression and relapse-free survival in breast cancer and HNSCC. *A*, miR-210 qPCR expression (DDCT) plotted as a function of ISCU mRNA expression (Illumina arrays) in the Oxford breast cancer series (*left*), and, relapse free survival for samples with high ISCU and low ISCU split by median in the Oxford breast cancer series (*right*). *B*, mRNA expression of the transcript hosting the miR-210 precursor plotted as a function of ISCU mRNA expression measure by Affymetrix U133b and U133a respectively in the Uppsala breast cancer series (*left*), and relapse free survival for samples with high ISCU and low ISCU split by median in the Uppsala breast cancer series (*right*). *C*, miR-210 qPCR expression (DDCT) plotted as a function of ISCU mRNA expression (Affymetrix arrays) in the Oxford-Manchester HNSCC cancer series where ISCU was initially found to be associated to hypoxia (*left*), and relapse free survival for samples with high ISCU and low ISCU split by median value in the Chung et al. HNSCC series (*right*).

The only data available for comparison of miR-210 with ISCU RNA in human tumour samples is from our breast and head and neck series. There was a highly significant inverse relationship of miR-210 to ISCU expression in 216 patients with breast cancer (rho = −0.39, p<0.001) (Figure 5A, *left*). There was a highly significant inverse correlation of ISCU with more aggressive high grade tumours (p = 0.008) and a poor relapse free survival (Figure 5A, *right*). In a multivariate analysis, ISCU remained significant (p = 0.028), along with nodes, grade and age (Supporting Table S2). In 2 other series of breast cancer (Rotterdam [26], 286 cases: Uppsala [27]; 235 cases) low ISCU was a poor prognostic factor in multivariate analysis (Supporting Tables S3 and S4) (p = 0.038 and 0.015 respectively). In the Uppsala series [27] we could assess the precursor miR-210 using the Affymetrix probes (Affymetrix U133b, 230710_at) and this also showed an inverse relationship of the precursor to ISCU and poor survival (Figure 5B). In our head and neck cancer series [25], there was an inverse relationship of these variables (Figure 5C, *left*). In a series with published follow up, Chung et al [28], there was a strong association of suppressed ISCU with poor outcome (Figure 5C, *right*). Analysis of expression in normal tissues versus tumour tissues in 9 tumour data sets using Oncomine showed in all studies suppression compared to normal tissues (Supporting Figure S4).

## Discussion

Our studies clearly show miR-210 regulates the expression of ISCU RNA and protein using the mimic in normoxia in cancer cell lines. ISCU mRNA and protein were suppressed under hypoxia and this effect was significantly reversed by anti-210. The incomplete reversal implies other mechanisms are also involved in ISCU downregulation and it is well recognised that RNA translation is reduced by hypoxia via the unfolded protein response [29].

A decrease in ISCU would impair the ability of cells to generate Fe-S clusters and would subsequently impact on the activity of enzymes that require these co-factor moieties. We showed that aconitase, a key enzyme of intermediary metabolism that requires Fe-S clusters, had decreased activity following ISCU reduction. Of specific relevance to cancer is the dual role of aconitase as IRP1 when it lacks its Fe-S cluster. IRP1 regulates the stability and translation of mRNA for transferrin and ferritin [24]. The effects of loss of aconitase would be to modify iron metabolism and increase its uptake, which is critical for tumour growth. Indeed, we observed an accumulation of intracellular iron upon transfection of cells with mimic-210 or siISCU.

Mutations in ISCU give rise to lactic acidosis in individuals after moderate exercise, providing strong evidence for the importance of this target in maintaining efficient Krebs cycle activity [30], [31]. An associated effect from decreased ISCU levels and subsequent decrease in the Krebs cycle activity was a switch to glycolysis and increased lactate production. This was induced in normoxia by miR-210 and thus is similar to the Warburg effect. This may be relevant in situations where miR-210 upregulation occurs in normoxia. For example, this has been shown in renal cancer cell lines with mutations in the von Hippel Lindau protein [14]. Conversely, in hypoxia, anti-210 reduced lactate production, reversing the Pasteur effect (switch to glycolysis in hypoxia).

Iron-sulfur clusters are integral for efficient activity of the mitochondrial electron transport chain. We have shown that Complex I activity is decreased under hypoxia and upon transfection of mimic-210 in normoxia. In addition to Complex I, Complexes II and III also contain Fe-S clusters and we speculate that the activities of all three complexes could be decreased in a miR-210 dependent manner. An impaired electron transport chain would lead to an increase in free radical production as a consequence of electron leakage. In particular, complex III is a major contributor to ROS in hypoxia [32] and complex I and II contribute about 30% of the ROS [33]. In normoxic conditions, transfection of mimic-210 led to increased free radical production. Furthermore, the normoxic ROS production observed with mimic-210 was almost completely prevented with a miR-210 resistant ISCU construct. Conversely, in hypoxia, we observed that the induction of miR-210 was associated with an increase in ROS, and this could be almost completely reversed by anti-210. This provides a major new link to explain the mechanism of ROS induction in hypoxia, which has been reported by several groups [33] and may account for the majority of hypoxic ROS induction. The ROS released in hypoxia have been shown to function as O_2_ sensors, acting as signalling agents that activate HIF [32], mediate tumour growth in vivo via a HIF dependent mechanism [5] and extend lifespan of cells [34].

In contrast to the hypoxic induction of ROS and alleviation with anti-210 observed in our study, Chan et al. could not measure any significant change in ROS production after exposure to hypoxia, and showed an inverted trend when miR-210 was inhibited [20]. The reason for this discrepancy is unclear but may potentially reflect an underlying difference in cancer compared to normal endothelial cells.

Finally, we analysed whether there was any association of ISCU downregulation in primary human cancers with hypoxia and outcome, and found an aggressive phenotype was associated with this change in studies of over 1000 patients, and downregulation compared to normal tissues in many tumour types.

In conclusion, we have found a major new mechanism for the response of cancer cells to hypoxia, co-ordinated by miR-210 suppression of ISCU and the subsequent decrease in activity of iron-sulfur cluster proteins (Supporting Figure S5). In addition to aconitase, many metabolic enzymes require Fe-S clusters for their activity suggesting that downregulation of ISCU would have far reaching consequences on cell metabolism. Mutations in many of these Fe-S cluster enzymes including succinate dehydrogenase subunits SDHD, SDHB and SDHC [35] and fumarate hydratase [36] are implicated in hyperproliferative disorders and cancer, demonstrating an important link between cellular metabolism and subsequent transformation and highlights a role for HIF, via a miR-210-ISCU axis in these processes. In addition to metabolic enzymes, several DNA repair enzymes [37] with Fe-S clusters are potentially targets. We have previously shown miR-210 can be detected at an elevated level in serum of cancer patients [38] so it will be of interest if they reflect the metabolic state of their primary cancer and hence could be used in future therapy selection. The regulation of distinct biological pathways by ISCU downregulation suggests a potential therapeutic approach of synthetic lethality whereby drugs that mediate DNA damage repaired by the iron-sulfur cluster containing helicases Rad3 [39] or Fanconi anaemia [37] could be use in synergy with PARP inhibitors, or inhibitors of glycolysis [36] and glutaminolysis in the subgroup of patients with highest miR-210 levels.

While this work was in preparation, three new miR-210 targets were validated, i.e. HOXA1, HOXA9 and FGFRL1, and the analysis of tumour xenografts derived from cancer cell lines overexpressing miR-210 suggested a potential involvement of miR-210 in tumour growth initiation [40].

## Materials and Methods

### Cell culture

Cell exposure to hypoxia (1%, or 0.1%, or 0.01% oxygen) was undertaken in a hypoxia incubator (MiniGalaxy A, RS Biotech, Scotland, UK), using a continuous flow of a humidified a mixture of 95% N_2_ and 5% CO_2_.

### miRNA mimic or inhibitor transfection

Transfection was performed with Dharmacon anti-210, miR-210 mimic, or control oligos (Thermo Scientific, CO, USA), at a final concentration of 20 nM, using Optimem serum-free medium and Oligofectamine reagent (both from Invitrogen, Paisley, UK) as per the manufacturer's protocol.

### Transfection and luciferase assays

Luciferase reporter plasmids containing 3′ untranslated regions (UTR) of ISCU or random genomic sequence (control) were obtained from SwitchGear Genomics (Menlo Park, CA. A Renilla luciferase expression plasmid (pRL-TK vector, Promega, Southampton, UK) was used as transfection control.

### Enzyme activity assays

Aconitase activity was using the Bioxytech Aconitase-340 assay (Oxis International Inc, Beverly Hills, CA). Data are presented as % of activity compared to control.

Cells were assayed for Complex I activity using the Mitoprofile Complex I Rapid Elisa Kit (MitoSciences, Eugene, OR, USA) according to manufacturer's instructions. Data are presented as % of activity compared to control.

### Lactate and pyruvate measurements

Lactate and pyruvate were assayed using kits from Instruchemie (Delfzijl, The Netherlands).

### MitoSOX staining

Mitochondrial superoxide was assayed using MitoSOX Red (Molecular Probes, Eugene, OR, USA). Images were acquired with a confocal microscope (Radiance 2000, Bio-Rad Laboratories, Hemel Hempstead, Herts, UK), and analysed as previously described [41].

### Gene expression data-mining

We previously derived a hypoxia metagene in primary head and neck cancer squamous cell carcinoma [HNSCC] [25]. Clusters of genes anti-correlated to miR-210 expression were formed as described previously [25]; Spearman correlation was used to identify genes significantly anti-correlated miR-210 and false discovery rate (FDR) was estimated using a permutation-based method [42]. Genes with FDR<0.05 were selected. We also used 5 target prediction algorithms:- TargetScanHuman (http://www.targetscan.org/); Pictar [43]; MiRanda (http://microrna.sanger.ac.uk/); DianaLab (http://diana.cslab.ece.ntua.gr/), miRDB (http://mirdb.org/miRDB/).

### Survival analyses and other statistical analyses

Spearman correlation was used for continuous variables and Wilcox test for categorical variables. The Log-Rank test was used for univariate, and Cox survival was used for multivariate analysis. Distant-relapse free survival (DRFS) and relapse-free survival (RFS) were calculated by the STEEP criteria [44]. Statistics were carried out using non-paired t-tests and significance is represented by: *, p<0.05, **, p<0.01, and ***, p<0.001.

## Supporting Information

Materials and Methods S1 (0.06 MB DOC)

Figure S1 Expression of miR-210 and ISCU in cancer cell lines. (A) In HCT116 cancer cells, miR-210 increases under hypoxia with the most robust induction seen at 48 hrs with 0.1% oxygen. (B) Under the same conditions, the strongest downregulation of ISCU mRNA is observed at 48 hrs with 0.1% oxygen. The expression levels of miR-210 and ISCU mRNA under hypoxia are relative to their normoxic expression levels at 24 hrs (N). Mean ± s.e.m. of three independent experiments is shown. (C, D) Knockdown of HIF1α but not HIF2α reverses the hypoxic suppression of ISCU mRNA at 48 hrs in MCF7 and HCT116 cell lines. Expression of ISCU mRNA is relative to scr in normoxia (N). Mean ± s.e.m. of three independent experiments is shown. (* p<0.05, ** p<0.01, *** p<0.001). (E) A panel of breast cancer cell lines were exposed to 1% oxygen for 24 hours (3 replicates/time point). miR-210 expression was measured by qPCR. Columns show mean fold difference in miR-210 expression between hypoxia and the parallel normoxia control samples using RNU48 as a reference; bars show s.e.m.. (F) ISCU was measured by qPCR comparing normoxia with cells grown in parallel under 1% oxygen; columns show downregulation under hypoxia, bars show s.e.m.
(1.01 MB TIF)

Figure S2 ISCU expression is regulated by miR-210. (A) Transfection of anti-210 partially rescues the hypoxic suppression of ISCU mRNA and mimic-210 overexpression decreases ISCU mRNA in HCT116 cells in normoxia at 48 hrs. Expression of ISCU mRNA is relative to the anti-ctrl in normoxia (N). Mean ± s.e.m. of two independent experiments for HCT116 is shown. (* p<0.05, ** p<0.01, *** p<0.001). (B) MCF7 cells were transfected with two siRNA against ISCU, siISCU1 and siISCU3. The ISCU protein normally seen under normoxic conditions is completely downregulated with both siRNAs. (C) Cellular subfractionation shows that mimic-210 downregulates both mitochondrial and cytosolic ISCU isoforms. Immunoblotting against Tom20 (Santa Cruz Biotechnologies, CA, USA) and actin were used as loading control of the mitochondrial and cytosolic compartment respectively. Cellular subfractionation was performed in MCF7 cells using the Mitochondrial Isolation Kit for cells in Culture (Thermofisher, CO, USA). (D, top panel) ISCU protein is downregulated in HCT116 lysates at 0.1% oxygen compared to normoxic lysates (N) when the anti-ctrl is transfected for 48 hrs. This decrease is partially rescued when the anti-210 is transfected. Under normoxic conditions, mimic-210 suppresses ISCU protein levels compared to mimic-ctrl transfected cells. (D, bottom panel) Quantification of the rescue of ISCU protein level upon transfection of anti-210. Transfection of anti-210 rescues the ISCU protein levels in HCT116 by approximately 40%. Mean ± S.E.M of two independent experiments is shown. (* p<0.05). (E) The 3′-UTR is required for the regulation of ISCU by miR-210. A luciferase reporter construct containing the full length 3′-UTR of ISCU or random sequence (Ctrl-3′-UTR) was co-transfected with 20 nM of mimic-210/mimic-ctrl or 40 nM of anti-210/anti-ctrl in MCF7 cells. The cotransfection of a vector expressing Renilla luciferase was used to normalize for transfection efficiency. The luciferase activity was expressed as a ratio of ISCU-3′-UTR to ctrl-3′-UTR relative to expression in normoxia. All experiments were carried out in triplicate, (n = 9); error bars denote the standard deviation. (***p<0.001). (F) MCF7 and HCT116 cells were transfected with an expression plasmid for a Myc-DKK tagged ISCU2 lacking the 3′-UTR. Subsequent transfection of siISCU3 reduced the protein levels of both endogenous and exogenous ISCU. Mimic-210 only affected the endogenous ISCU protein level.
(1.01 MB TIF)

Figure S3 Iron uptake assessed by ferric iron staining. Knockdown of ISCU with siISCU3 led to an accumulation of ferric iron compared to control siRNA transfected HCT116 cells. This effect was reproduced when cells were transfected with mimic-210 compared to mimic-ctrl. HCT116 cells were transfected as described above and treated with 100 mg/L Ferric Ammonium Citrate for 16 hours. The cells were then fixed and stained with 4% Formalin and Perl's solution (1% K4Fe(CN)6 and 1% HCl) for 30 minutes at room temperature. Cells were then incubated with 0.75 mg/ml diaminobenzidine (DAB), H2O2 in 1 M Tris pH 7.5 for 60 minutes. The reaction was then completed by washing the cells in PBS. Original magnification: 100×.
(1.19 MB TIF)

Figure S4 Oncomine data. The Oncomine website (Oncomine.org) was searched for microarrays containing the gene ISCU. When compared to normal tissue, ISCU was significantly downregulated in 9 tumour microarray experiments; data for relative expression units, normalised to median z-score to enable comparison across multiple studies, were downloaded and graphed. p<0.05 for all, Student's t-test.
(0.97 MB TIF)

Figure S5 Hypoxia regulates ISCU via HIF1α induction of miR-210. miR-210 represses ISCU 3′-UTR and reduces ISCU protein. Reduced Fe-S assembly in the mitochondrial electron transport chain results in inhibition of major sites of electron transfer. This is a key mechanism for generating ROS, which have both positive and negative effects in hypoxia. Reduction of aconitase activity inhibits the Krebs cycle, resulting in an increase in glycolysis and lactate production. Aconitase 1 (cytosolic aconitase) when depleted of Fe-S becomes an iron regulatory protein, irp1, binding to iron response elements in the 3′-UTR of the transferrin receptor. This stabilises the mRNA increasing transcription and enhancing iron uptake. Other Fe-S proteins are potential targets.
(0.60 MB TIF)

Table S1 Top predicted mir-210 targets using tumour data plus algorithms.
(0.05 MB DOC)

Table S2 Multivariate analysis of ISCU expression and relapse free survival in the Oxford: breast cancer series [14] (N = 216).
(0.05 MB DOC)

Table S3 Multivariate analysis of mRNA ISCU expression and disease-specific survival in the Uppsala breast cancer series published series [27] (N = 235).
(0.05 MB DOC)

Table S4 Multivariate analysis of ISCU expression and relapse free survival in the Rotterdam series of lymph-node-negative primary breast cancer [26] (N = 286).
(0.05 MB DOC)
