# Supplementary material for: MicroRNA-210 Regulates Mitochondrial Free Radical Response to Hypoxia and Krebs Cycle in Cancer Cells by Targeting Iron Sulfur Cluster Protein ISCU
Source: PLoS One. 2010 Apr 26;5(4):e10345. doi: 10.1371/journal.pone.0010345 (PMC2859946; doi:10.1371/journal.pone.0010345)
Supplement: Materials and Methods S1 — (0.06 MB DOC) [file pone.0010345.s001.doc]

**A consensus score to select targets**

To select potential targets we used 5 of the published main target prediction algorithms and combined then with the hypoxia downregulated metagene and the miR-210 down-regulated cluster signature. The potential targets produced by these algorithms and data-mining were annotated to a common ID, Ensembl Ids, using BioMaRt (http://www.biomart.org/). For each target, its presence in a given list was scored using a non-parametric score between 0 and 1. This score was either binary (0=absent, 1=present) or continous (ranks from 0=absent, to 1=highest ranked gene) depending on the degree of “conservativness” of the initial list/algorithm. Specifically, with respect to MiRanda and microT predictions, transcripts were ranked by the algorithm score from 1 (highest score) to 0 (if not present). With respect to TargetScan, miRDB and Pictar, transcripts were ranked as 1 if present in the list and 0 if not present. Transcripts in the hypoxia metagene were ranked using the order published in the original paper, that is ordered by decreasing number of clusters in which the genes were found [1], from 1 (genes shared by many clusters) to 0 (genes not present in the signature). Finally, transcripts in the miR-210 down-regulated cluster were ranked by the FDR, from 1 (lowest FDR) to 0 (transcript not in the signature).

For each transcript, a mean rank score was calculated including the ranking of the 5 databases, the hypoxia metagene and the miR-210 cluster. This mean score was used to rank the transcripts from highest, very likely to be a target (maximum is 1=top score by all predictions and signatures) to lowest, that is very unlikely to be a target (minimum is 0=absent from any list).

### RNA extraction

Cells were lysed in Tri Reagent (Sigma-Aldrich), and extracted using ethanol precipitation. RNA quality and quantity were confirmed using the NanoDrop ND-1000 spectrophotometer (Wilmington, USA).

### Real-time reverse transcription PCR

MiRNA expression was assessed by real-time PCR according to the TaqMan MicroRNA Assay protocol (Applied Biosystems, Warrington, UK). RT-PCR for miR-210 in tumours has been described previously [2] . Relative miR-210 expression was determined by the 2^(-DDCt)^ method [3], normalising to the small nRNA RNU43, RNU44, or RNU48.

### Western blots

Primary antibodies to ISCU (Novus Biologicals, CO, USA), HIF1α (BD Transduction Laboratories, KY, USA), and ß-actin (Sigma-Aldrich) were used and the blots were visualised using ECL reagent (GE Healthcare, Buckinghamshire, UK).

**Gene Silencing by RNA interference**

Transfections of siRNA duplexes targeting HIF1α, HIF2α, ISCU (siISCU1 and 3), and a scramble control (scr) at a final concentration of 20 nM, were performed in Optimem (Invitrogen), using Oligofectamine reagent (Invitrogen). Details of the oligonucleotides (purchased from Eurogentec, Southampton, UK) are as follows - HIF1α antisense: 5'-CUGAUGACCAGCAACUUGAdTdT-3' and HIF1α sense: 5'-UCAAGUUGCUGGUCAUCAGdTdT-3'; HIF2α antisense: 5’-UUCAAUCUUCAGGUCGUUAdTdT-3' and HIF2α sense: 5’-UAACGACCUGAAGAUUGAAdTdT-3’; ISCU1 antisense: 5’-UUUCCUUUCACCCAUUCAGdTdT-3' and ISCU1 sense 5’-CUGAAUGGGUGAAAGGAAAdTdT-3’; ISCU3 antisense: 5’-AAUUUCAUUACGUCACCACdTdT-3' and ISCU3 sense: 5’-GUGGUGACGUAAUGAAAUUdTdT-3’; scr antisense 5'-ACGACACGCAGGUCGUCAUdTdT-3' and scr sense: 5'-AUGACGACCUGCGUGUCGUdTdT-3’

**Plasmid transfection**

Cells were transfected with a plasmid overexpressing a C-terminal Myc-DKK tagged ISCU2, pCMV6-ISCU2, (OriGene Technologies, Inc, Rockville, MD, USA), using Lipofectamine 2000 (Invitrogen) and siRNA or miRNA mimic/anti-miR toaccording to manufacturer’s instructions. For the ROS rescue experiments, cells were co-transfected with pcDNA3-GFP for co-localisation.

**Annexin V flow cytometry**

Cells were harvested with trypsin, and stained with Annexin V Alexa Fluor 647 conjugate and propidium iodide (PI) (Invitrogen,UK). Cells were analyzed in a FACS analyzer Cyan ADP (Dako, Glostrup, Denmark).

###### Colony Assays

Cells were reverse transfected using the manufacturer’s protocol for siPORT *NeoFX* transfection agent (Applied Biosystems), with 50 nM of either a locked nucleic acid probe for miR-210 (Exiqon product number 118103-00; sequence, 5'-TCAGCCGCTGTCACACGCACAG-3') or negative control probe (Exiqon product number 199002-00; sequence, 5'-GTGTAACACGTCTATACGCCCA-3'). 24h after transfection the medium was changed and the cells were placed under normoxia or hypoxia (0.1 % or 0.01% oxygen) for 72 hrs. Colonies were counted 12 days later, and surviving fraction of cells was determined as described in [4].

### Xenografts

Animal work was performed with appropriate ethical approval. SCID mice were implanted subcutaneously with U87 glioblastoma cells. Animals received either bevacizumab, (10 mg/kg, Genentech, CA, USA) every 3 days from day 12 or saline control.

**REFERENCES**

1. Winter SC, Buffa FM, Silva P, Miller C, Valentine HR, et al. (2007) Relation of a hypoxia metagene derived from head and neck cancer to prognosis of multiple cancers. Cancer Res 67: 3441-3449.

2. Camps C, Buffa FM, Colella S, Moore J, Sotiriou C, et al. (2008) hsa-miR-210 Is Induced by Hypoxia and Is an Independent Prognostic Factor in Breast Cancer. Clin Cancer Res 14: 1340-1348.

3. Vandesompele J, De Preter K, Pattyn F, Poppe B, Van Roy N, et al. (2002) Accurate normalization of real-time quantitative RT-PCR data by geometric averaging of multiple internal control genes. Genome Biol. pp. RESEARCH0034.

4. Crosby ME, Jacobberger J, Gupta D, Macklis RM, Almasan A (2007) E2F4 regulates a stable G2 arrest response to genotoxic stress in prostate carcinoma. Oncogene 26: 1897-1909.
