# Supplementary figures and images for: MicroRNA-210 Regulates Mitochondrial Free Radical Response to Hypoxia and Krebs Cycle in Cancer Cells by Targeting Iron Sulfur Cluster Protein ISCU
Source: PLoS One. 2010 Apr 26;5(4):e10345. doi: 10.1371/journal.pone.0010345 (PMC2859946; doi:10.1371/journal.pone.0010345)

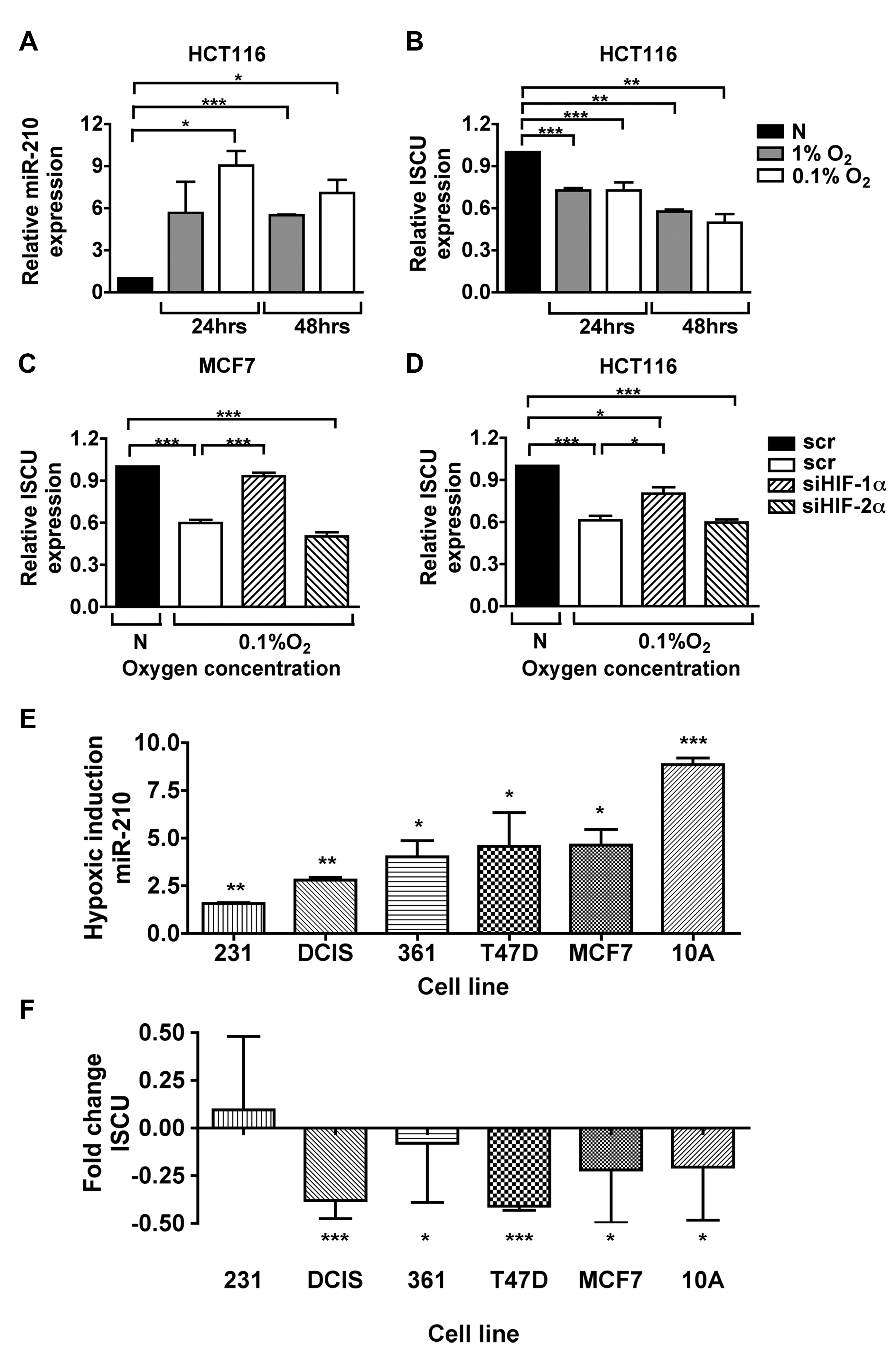

Supplement: Figure S1 — Expression of miR-210 and ISCU in cancer cell lines. (A) In HCT116 cancer cells, miR-210 increases under hypoxia with the most robust induction seen at 48 hrs with 0.1% oxygen. (B) Under the same conditions, the strongest downregulation of ISCU mRNA is observed at 48 hrs with 0.1% oxygen. The expression levels of miR-210 and ISCU mRNA under hypoxia are relative to their normoxic expression levels at 24 hrs (N). Mean ± s.e.m. of three independent experiments is shown. (C, D) Knockdown of HIF1α but not HIF2α reverses the hypoxic suppression of ISCU mRNA at 48 hrs in MCF7 and HCT116 cell lines. Expression of ISCU mRNA is relative to scr in normoxia (N). Mean ± s.e.m. of three independent experiments is shown. (* p<0.05, ** p<0.01, *** p<0.001). (E) A panel of breast cancer cell lines were exposed to 1% oxygen for 24 hours (3 replicates/time point). miR-210 expression was measured by qPCR. Columns show mean fold difference in miR-210 expression between hypoxia and the parallel normoxia control samples using RNU48 as a reference; bars show s.e.m.. (F) ISCU was measured by qPCR comparing normoxia with cells grown in parallel under 1% oxygen; columns show downregulation under hypoxia, bars show s.e.m. (1.01 MB TIF) [file pone.0010345.s002.tif]

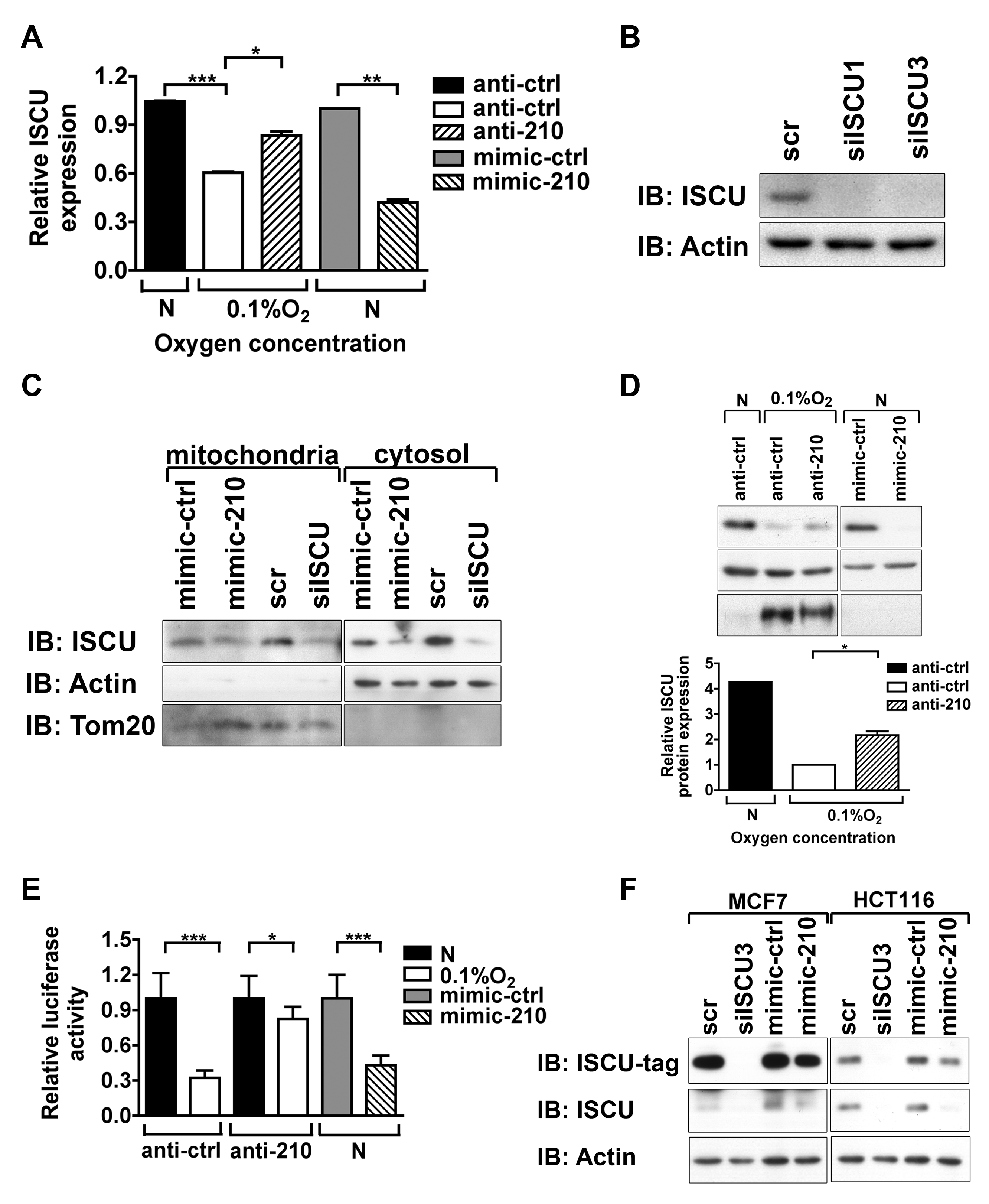

Supplement: Figure S2 — ISCU expression is regulated by miR-210. (A) Transfection of anti-210 partially rescues the hypoxic suppression of ISCU mRNA and mimic-210 overexpression decreases ISCU mRNA in HCT116 cells in normoxia at 48 hrs. Expression of ISCU mRNA is relative to the anti-ctrl in normoxia (N). Mean ± s.e.m. of two independent experiments for HCT116 is shown. (* p<0.05, ** p<0.01, *** p<0.001). (B) MCF7 cells were transfected with two siRNA against ISCU, siISCU1 and siISCU3. The ISCU protein normally seen under normoxic conditions is completely downregulated with both siRNAs. (C) Cellular subfractionation shows that mimic-210 downregulates both mitochondrial and cytosolic ISCU isoforms. Immunoblotting against Tom20 (Santa Cruz Biotechnologies, CA, USA) and actin were used as loading control of the mitochondrial and cytosolic compartment respectively. Cellular subfractionation was performed in MCF7 cells using the Mitochondrial Isolation Kit for cells in Culture (Thermofisher, CO, USA). (D, top panel) ISCU protein is downregulated in HCT116 lysates at 0.1% oxygen compared to normoxic lysates (N) when the anti-ctrl is transfected for 48 hrs. This decrease is partially rescued when the anti-210 is transfected. Under normoxic conditions, mimic-210 suppresses ISCU protein levels compared to mimic-ctrl transfected cells. (D, bottom panel) Quantification of the rescue of ISCU protein level upon transfection of anti-210. Transfection of anti-210 rescues the ISCU protein levels in HCT116 by approximately 40%. Mean ± S.E.M of two independent experiments is shown. (* p<0.05). (E) The 3′-UTR is required for the regulation of ISCU by miR-210. A luciferase reporter construct containing the full length 3′-UTR of ISCU or random sequence (Ctrl-3′-UTR) was co-transfected with 20 nM of mimic-210/mimic-ctrl or 40 nM of anti-210/anti-ctrl in MCF7 cells. The cotransfection of a vector expressing Renilla luciferase was used to normalize for transfection efficiency. The luciferase activity was expresse [file pone.0010345.s003.tif]

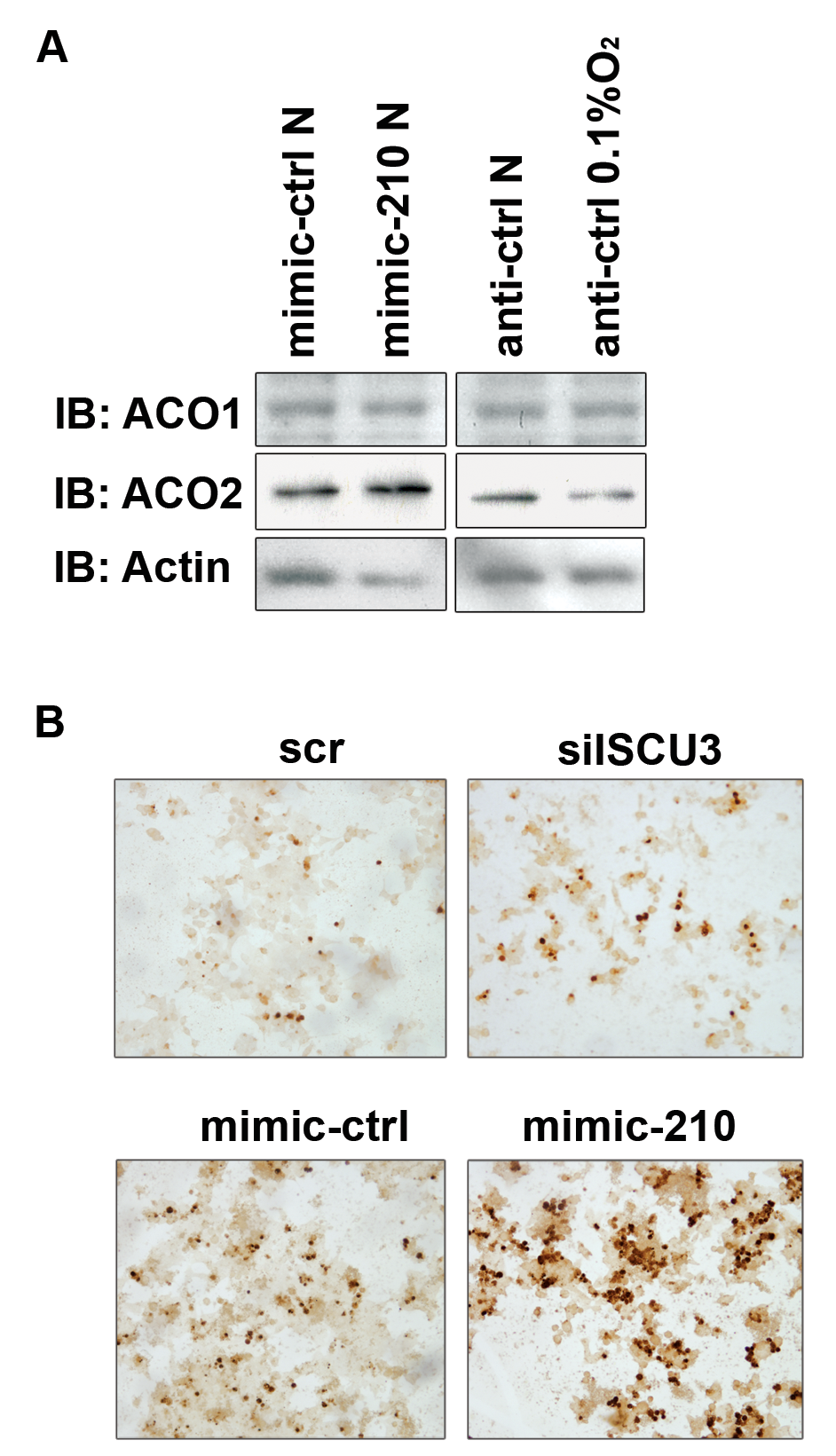

Supplement: Figure S3 — Iron uptake assessed by ferric iron staining. Knockdown of ISCU with siISCU3 led to an accumulation of ferric iron compared to control siRNA transfected HCT116 cells. This effect was reproduced when cells were transfected with mimic-210 compared to mimic-ctrl. HCT116 cells were transfected as described above and treated with 100 mg/L Ferric Ammonium Citrate for 16 hours. The cells were then fixed and stained with 4% Formalin and Perl's solution (1% K4Fe(CN)6 and 1% HCl) for 30 minutes at room temperature. Cells were then incubated with 0.75 mg/ml diaminobenzidine (DAB), H2O2 in 1 M Tris pH 7.5 for 60 minutes. The reaction was then completed by washing the cells in PBS. Original magnification: 100×. (1.19 MB TIF) [file pone.0010345.s004.tif]

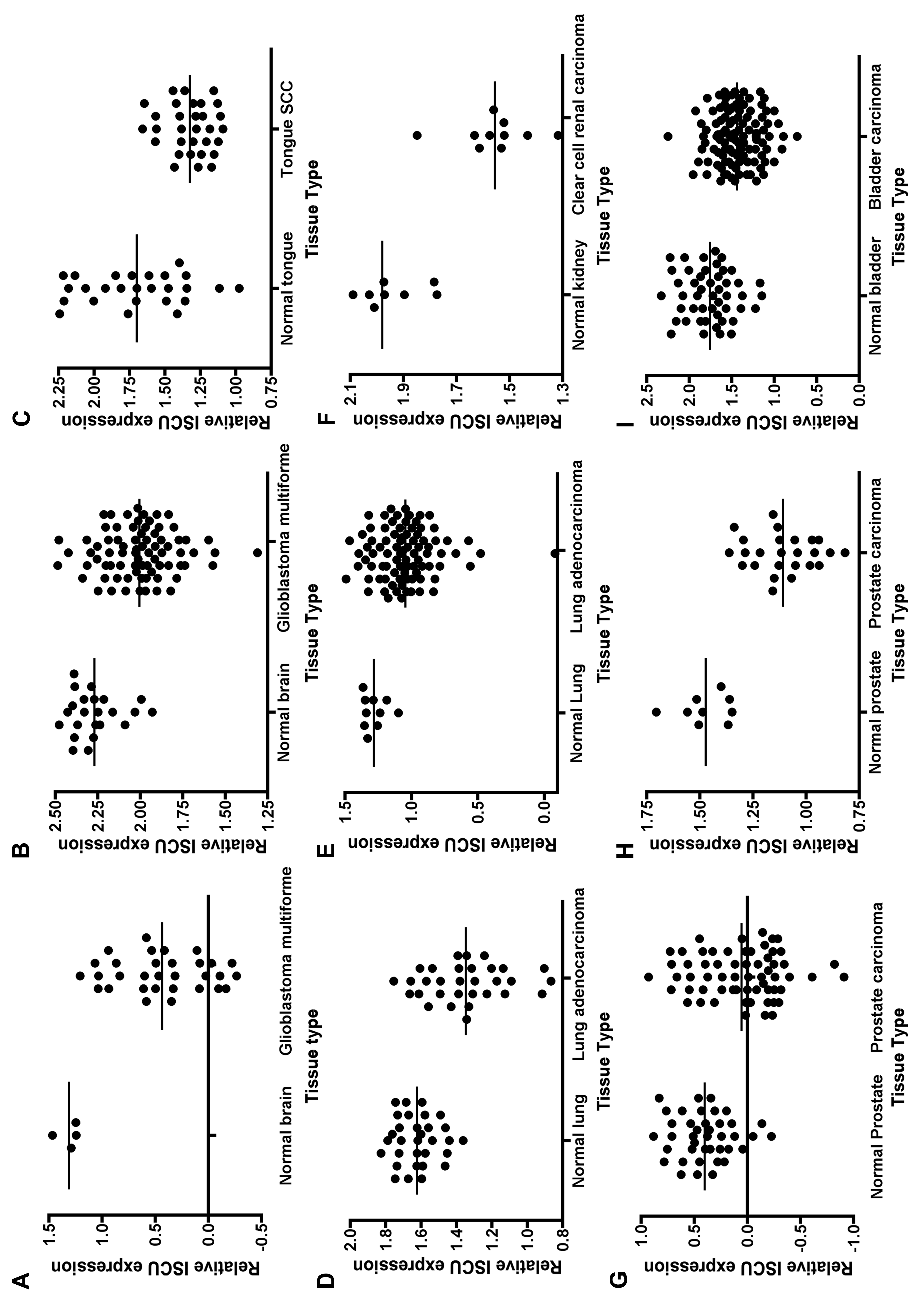

Supplement: Figure S4 — Oncomine data. The Oncomine website (Oncomine.org) was searched for microarrays containing the gene ISCU. When compared to normal tissue, ISCU was significantly downregulated in 9 tumour microarray experiments; data for relative expression units, normalised to median z-score to enable comparison across multiple studies, were downloaded and graphed. p<0.05 for all, Student's t-test. (0.97 MB TIF) [file pone.0010345.s005.tif]

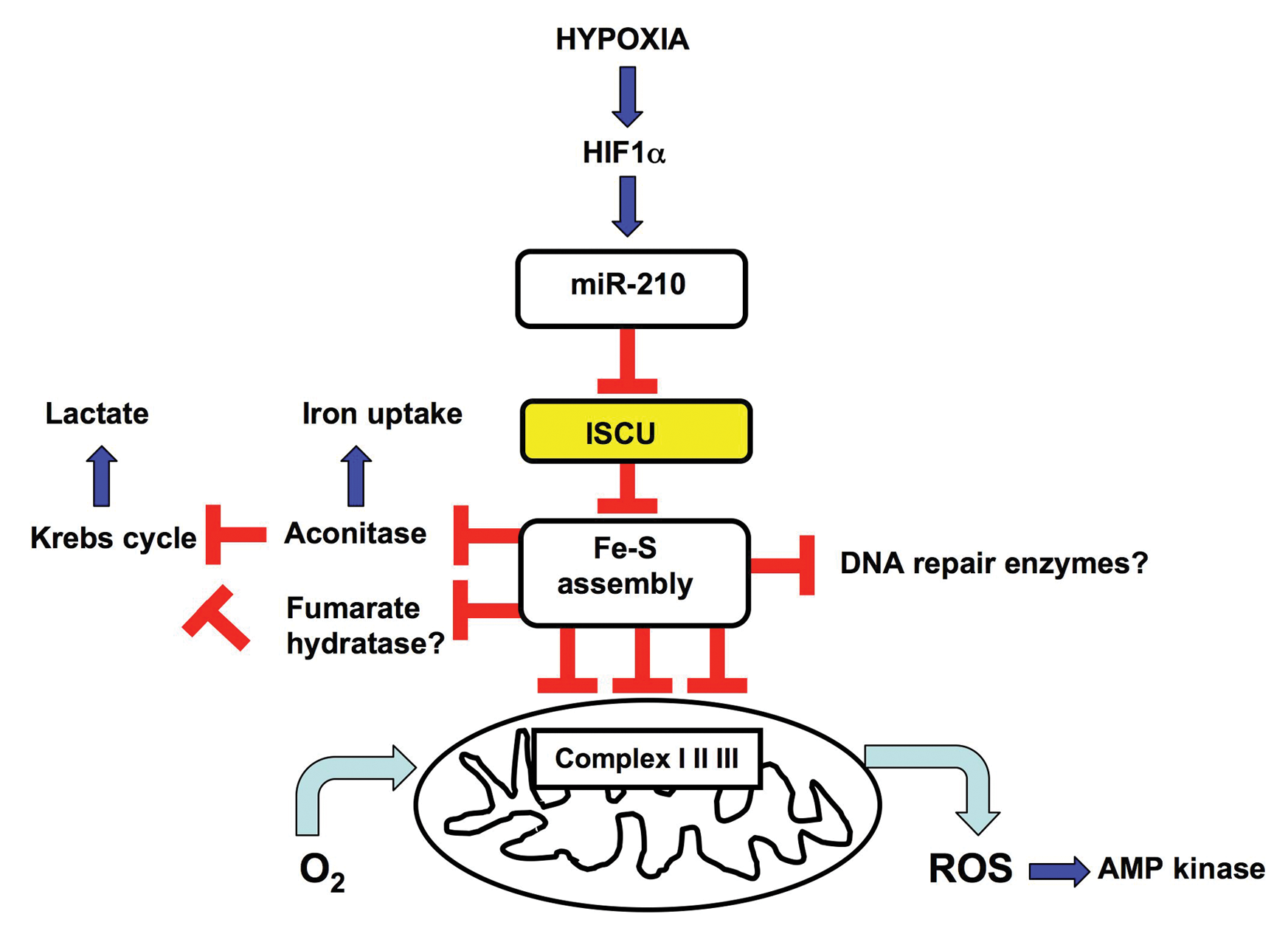

Supplement: Figure S5 — Hypoxia regulates ISCU via HIF1α induction of miR-210. miR-210 represses ISCU 3′-UTR and reduces ISCU protein. Reduced Fe-S assembly in the mitochondrial electron transport chain results in inhibition of major sites of electron transfer. This is a key mechanism for generating ROS, which have both positive and negative effects in hypoxia. Reduction of aconitase activity inhibits the Krebs cycle, resulting in an increase in glycolysis and lactate production. Aconitase 1 (cytosolic aconitase) when depleted of Fe-S becomes an iron regulatory protein, irp1, binding to iron response elements in the 3′-UTR of the transferrin receptor. This stabilises the mRNA increasing transcription and enhancing iron uptake. Other Fe-S proteins are potential targets. (0.60 MB TIF) [file pone.0010345.s006.tif]
