# Supplementary material for: MicroRNA-210 Regulates Mitochondrial Free Radical Response to Hypoxia and Krebs Cycle in Cancer Cells by Targeting Iron Sulfur Cluster Protein ISCU
Source: PLoS One. 2010 Apr 26;5(4):e10345. doi: 10.1371/journal.pone.0010345 (PMC2859946; doi:10.1371/journal.pone.0010345)
Supplement: Table S1 — Top predicted mir-210 targets using tumour data plus algorithms. (0.05 MB DOC) [file pone.0010345.s007.doc]

| **Ensemble** | **Symbol** | **Target**  **Scan** | **Pictar** | **miRanda** | **microT** | **miRDB** | **miR210 cluster breast** | **miR210 cluster HNSCC** | **Down-reg. hypoxia signature** | **Overall**  **Rank**  **Score** |
| --- | --- | --- | --- | --- | --- | --- | --- | --- | --- | --- |
| **136003** | **ISCU** | **1** | **0** | **0.03** | **0.25** | **1** | **0.73** | **0.98** | **0.13** | **0.52** |
| **143590** | **EFNA3** | **1** | **1** | **0.65** | **0.62** | **0** | **0.00** | **0.00** | **0.00** | **0.41** |
| **136960** | **ENPP2** | **0** | **0** | **0.69** | **0.00** | **0** | **0.73** | **0.87** | **0.78** | **0.38** |
| **171246** | **NPTX1** | **0** | **1** | **0.87** | **0.90** | **0** | **0.00** | **0.00** | **0.00** | **0.35** |
