# Supplementary material for: MicroRNA-210 Regulates Mitochondrial Free Radical Response to Hypoxia and Krebs Cycle in Cancer Cells by Targeting Iron Sulfur Cluster Protein ISCU
Source: PLoS One. 2010 Apr 26;5(4):e10345. doi: 10.1371/journal.pone.0010345 (PMC2859946; doi:10.1371/journal.pone.0010345)
Supplement: Table S2 — Multivariate analysis of ISCU expression and relapse free survival in the Oxford: breast cancer series [14] (N = 216). (0.05 MB DOC) [file pone.0010345.s008.doc]

| Variable | HR | 95.0% CI | | p-value |
| --- | --- | --- | --- | --- |
|  |  | Lower | Upper |  |
| ISCU suppression | 2.6 | 1.11 | 6.1 | 0.03 |
| Age (decade) | 1.4 | 1.13 | 1.8 | .003 |
| Node number | 1.2 | 1.14 | 1.3 | .000 |
| Grade | 1.6 | 1.13 | 2.3 | .009 |

Reduced Model after Backward Stepwise Likelihood Selection; only variables with p<0.05 were retained in the final model. Initial variables included in the model: ISCU suppression (continuous mRNA expression ranked from high to low and normalised between 0 and 1), ER status, Tumour size, Positive Lymph Node Number, Age, Grade, Tamoxifen (yes, no).
