# Supplementary material for: MicroRNA-210 Regulates Mitochondrial Free Radical Response to Hypoxia and Krebs Cycle in Cancer Cells by Targeting Iron Sulfur Cluster Protein ISCU
Source: PLoS One. 2010 Apr 26;5(4):e10345. doi: 10.1371/journal.pone.0010345 (PMC2859946; doi:10.1371/journal.pone.0010345)
Supplement: Table S3 — Multivariate analysis of mRNA ISCU expression and disease-specific survival in the Uppsala breast cancer series published series [27] (N = 235). (0.05 MB DOC) [file pone.0010345.s009.doc]

| Variable | HR | 95.0% CI | | p-value |
| --- | --- | --- | --- | --- |
|  |  | Lower | Upper |  |
| ISCU suppression | 3.26 | 1.26 | 8.47 | 0.015 |
| Size (mm) | 1.04 | 1.01 | 1.06 | 0.002 |
| Nodal Status | 2.87 | 1.61 | 5.11 | 0.000 |

Reduced Model after Backward Stepwise Likelihood Selection; only variables with p<0.05 were retained in the final model. Initial variables included in the model were ISCU suppression (continuous mRNA expression ranked from high to low and normalised between 0 and 1) and available clinical variables: ER status, PgR status, Tumour size, Lymph Node status (0=neg, 1=pos), Age, Grade
