# Supplementary material for: MicroRNA-210 Regulates Mitochondrial Free Radical Response to Hypoxia and Krebs Cycle in Cancer Cells by Targeting Iron Sulfur Cluster Protein ISCU
Source: PLoS One. 2010 Apr 26;5(4):e10345. doi: 10.1371/journal.pone.0010345 (PMC2859946; doi:10.1371/journal.pone.0010345)
Supplement: Table S4 — Multivariate analysis of ISCU expression and relapse free survival in the Rotterdam series of lymph-node-negative primary breast cancer [26] (N = 286). (0.05 MB DOC) [file pone.0010345.s010.doc]

| Variable | HR | 95.0% CI | | p-value |
| --- | --- | --- | --- | --- |
|  |  | Lower | Upper |  |
| ISCU suppression | 2.01 | 1.04 | 3.87 | 0.038 |

Reduced Model after Backward Stepwise Likelihood Selection; only variables with p<0.05 were retained in the final model. Initial variables included in the model were ISCU suppression (continuous mRNA expression ranked from high to low and normalised between 0 and 1) and available clinical variables: ER status, Lymph Node status (0=neg, 1=pos)
